# The virus made me lose control: The impact of COVID-related work changes on employees’ mental health, aggression, and interpersonal conflict

**DOI:** 10.3389/fpubh.2023.1119389

**Published:** 2023-04-11

**Authors:** Changlin Han, Ruyi Zhang, Xiyao Liu, Xueling Wang, Xiaotong Liu

**Affiliations:** ^1^School of Business, Qingdao University, Qingdao, Shandong, China; ^2^Student Affairs Department, Shandong University, Jinan, Shandong, China

**Keywords:** COVID-related work changes, ego depletion, mental health, interpersonal conflict, aggression, trait resilience

## Abstract

**Introduction:**

COVID-related work changes have seriously disrupted employees’ familiar routines and hampered their lives and work. Although this topic has drawn rising attention, to our knowledge, limited studies have investigated the impact of COVID-related work changes on employees’ mentality and behavior. In this paper, we developed a moderated mediation model based on ego depletion theory to test how and when COVID-related work changes impact employees’ mental health, interpersonal conflict, and aggression behavior.

**Methods:**

We collected 536 valid participants by conducting a questionnaire survey in a large Chinese manufacturing company, and tested our proposed theoretical model and hypotheses using SPSS 26.0 and Mplus 8.1.

**Results:**

The empirical results showed that COVID-related work changes would harm employees’ mental health and boost their interpersonal conflict and aggression via increasing their ego depletion. Moreover, trait resilience has an intervention in the relationship between COVID-related work changes and employees’ ego depletion, which weakens the indirect impact of COVID-related work changes on mental health, interpersonal conflict, and aggression.

**Discussion:**

These findings suggest that although COVID-related work changes were inevitable, managers should take measures to improve the employees’ mental status and avoid conflicts promptly while taking steps to keep organizations on track.

## Introduction

The outbreak of the COVID-19 pandemic brings changes to the market, which have posed significant challenges to organizations’ daily operations(e.g., workplace lockdowns and mandatory quarantine) (1). In response to those challenges, organizations have to implement arrangements to ensure regular company operations while limiting the spread of the viruses, such measures including downsizing (2), mergers, and restructuring (3, 4). Therefore, employees were forced to adapt to a new and flexible working environment, which reflects employees’ work changes highly related to their organizational restructuring. For instance, employees might experience company relocation, remote work forms, and changes in job contracts (5). Also, different from the work content and procedures in the past, employees had to confront more internet-related additional job demands (6) and complex work situations, such as reduced shifts, paid or unpaid temporary leave, quarantined or self-isolated (7). In fact, these changes are unavoidable. COVID-related work changes have seriously disrupted employees’ familiar routines and negatively influenced their psychological as well as behavioral performance. For example, previous studies have confirmed that some of these changes are closely related to employees’ emotional exhaustion (8), burnout (9), a decline in work engagement (10), and decreased psychological well-being and productivity (11).

Although the negative effect of COVID-related work changes on employees has drawn rising attention, there are questions that remain to be explored. First, the comprehensive impact of COVID-related work changes on employees’ mental health and deviant workplace behavior has not been thoroughly discussed. Most studies on the impact of COVID-related work changes on employees’ psychological state and behavior have looked into a specific aspect of work changes, such as changes in working characteristics (i.e., decreased physical activity, lack of communication with coworkers) (12) or workplace adjustment (i.e., working from home, workplace redesign) (13, 14). Hence, it is unclear whether COVID-related work changes impact employee positively or negatively from a broad concept, given that the evidence from the existing literature is inadequate. Second, there has been little research on the mediating mechanisms of the relationship between COVID-related work changes and employees’ mental health and deviant workplace behavior. Previous scholars have mainly focused on the concept of work concerns to explain the impact of COVID-related work changes on employees’ mental health and negative behavior (15–17). Furthermore, it is unclear whether the effects of COVID-related work changes on mental health and deviant workplace behaviors vary across individuals with different trait resilience. Even though individual trait resilience has been identified as a protective factor buffering the impacts of risk factors under challenging situations (18, 19).

**Figure 1:**
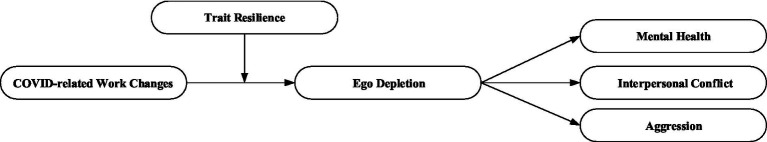
Theoretical model.

In this study, we develop a moderated mediation model to investigate how and when COVID-related work changes may influence employees’ mental health and deviant workplace behavior using ego depletion theory. Specifically, we choose two typical types of workplace deviance, interpersonal conflict and aggression, which are prevalent problems in organizations and seriously damage the well-being of others (20–22). According to ego depletion theory, ego depletion can be characterized as a state that manifests as a reduction of self-capacity or willingness to engage in volitional action caused by a prior exercise of volition (23). In this vein, we assume that employees need to spend plenty of self-control resources to overcome the difficulties and challenges posed by the COVID-related work changes, which can put them in a state of self-depletion. Further, employees with depleted resources are less likely to be able to regulate their negative emotions and aggressive impulses, which leads to decreased mental health but raises engagement in workplace deviant behavior. In addition, as suggested by the ego-depletion theory, individuals with different personalities react differently to resource depletion. Hence, we also consider trait resilience, the ability to bounce back or recover from stress (24, 25), as an individual’s difference and examine its moderating effect on the relationship between COVID-related work changes and employee ego depletion. The theoretical model is shown in Figure 1.

This research contributes to the existing literature in three ways. First, we expand on work changes literature by shedding light on the effect of COVID-related work changes on employees’ mental health and deviant workplace behavior. Although many previous researchers have examined the relationship between changes in a specific aspect of work and employee positive or negative responses during COVID-19 (9, 26, 27), we explore the comprehensive influence of COVID-related work changes on employees’ mental health and deviant workplace behavior. Examining the correlation also extends our knowledge of the antecedents of employees’ mental health and deviant workplace behavior. Second, we reveal an intermediate link accounting for the complete process of how COVID-related work changes are associated with mental health and deviant workplace behavior. Such contribution disclose ego depletion as an essential factor in bridging the COVID-related work changes and employee mentality and workplace behavior, thus providing scholars with a deeper understanding of the root cause of their relationship. Third, our study extends the moderating mechanism of the influence of COVID-related work changes on employee mental health and deviant workplace behavior. It is worth mentioning that no specific studies concern the role of resilience factors in the relationship between the COVID-19 outbreak and employees’ outcomes, regardless of existing research points to the importance of further exploring the role of trait resilience as a protective factor for one’s mental health during the COVID-19 crisis (28). Therefore, our study’s contribution lies in extending the concept of trait resilience as it has been applied to literature in the current study.

## Theory and hypotheses

### COVID-related work changes and ego depletion

COVID-19 brings drastic changes in external employment circumstances that employees need to face. In fact, the pandemic has led to dramatic economic dislocation and disruption in the work process (1), so most corporations suffer from demand–supply-production interruptions (29), which lead to downsizing. The dramatic reduction in labor demand puts employees at great risk of losing their jobs while other job opportunities become scarce (30). Besides coping with the pressures brought by the deterioration of the labor market, employees also need to make adjustments in the face of alternative work arrangements, which provide additional learning challenges. As such, employees are required to be familiar with the new work environment and quickly adapt to various internet technology (31, 32).

Throughout history, people have shown an extraordinary ability to regulate themselves and control impulses. The ability to self-control enables individuals to engage in goal-oriented behavior and achieve ideal long-term results (33). In line with the definition of Baumeister et al. (23), who first put forward the concept of ego depletion, they pointed out that individuals consume their limited resources in the process of self-control. Mental resources such as energy or power are consumed in the process of regulating themselves by coping with stress, regulating emotions, and resist temptation (34). Once such resources are depleted, individuals will fall into a state called “ego depletion” (23) and then misbehave (35).

This paper argues that COVID-related work changes are positively related to ego depletion. First, according to ego depletion theory, individuals may experience ego depletion because of the excessive consumption of self-regulating resources (23). As mentioned above, stress coping and emotion regulation are the two main channels individuals consume self-regulatory resources, an unavoidable experience during a pandemic. To get through the hardship of the pandemic, employees need to grit their teeth and adapt to the stressful work environment, changeable work arrangement, and unfamiliar work characteristics. The novelty experiences force employees to engage in more self-regulation activities than past, eventually leading to an overconsumption of employees’ limited mental resources. Second, ego depletion theory indicates that actions against personal willingness considerably consume self-control resources (23). As new management systems are issued due to COVID-related work changes, companies usually have more restrictions on employees’ daily work schedules, such as travel constraints and job deadline constraints. Even though these measurements benefit companies, comparatively, employees’ work autonomy has declined. Employees have to familiarize themselves with the new regulations in order to adjust their behavior to new rules. As a result, due to the loss of autonomy in decision-making and actions, employees would engage in more behaviors against their own will and hence fall into a state of ego depletion. Therefore, the following hypothesis is proposed:

*H1*: COVID-related work changes are positively related to ego depletion.

### Ego depletion and mental health

According to the World Health Organization (36), mental health is a state of well-being in which the individual realizes his or her abilities to cope with the normal stresses of life, can work productively and fruitfully and can contribute to his or her community.

Previous studies have shown that employees’ mental health is closely related to their work attitude (37, 38) and ultimately affects their work performance (39). However, with the outbreak of the pandemic, the overall mental health level of employees has shown a downward trend (40), which has attracted increasing attention from scholars. For example, Gabriel et al. (41) showed that COVID-19 increased employees’ job burnout by aggravating the consumption of work resources, ultimately reducing their work performance.

In this paper, we argue that the state of ego depletion is harmful to employees’ mental health. First, based on ego depletion theory, individuals need to consume self-control resources in controlling emotion (23, 42). However, when employees encounter ego depletion, self-control resources are lost (23). Due to limited mental resources, individuals could not regulate their emotions as usual. Thereby, the negative emotions accumulate and ultimately damage their mental health. Second, experiencing ego depletion normally leads to individual cognitive bias, which has been manifested by studies that lead to underestimation of their ability to control the external environment and having more pessimistic expectations for the future (43). Such harmful impacts are not temporary but rather a long-term and ongoing process. Suppose individuals stay in a negative psychological state for the long term without any other psychological support. In that case, psychological problems such as depression (44), anger (45), anxiety (46), and burnout (47) will be induced and, in turn, seriously damage the individual’s mental health. Therefore, we propose the following hypothesis:

*H2a*: Ego depletion is negatively related to mental health.

### Ego depletion, interpersonal conflict, and aggression

Interpersonal conflict and aggression are two common types of deviant workplace behaviors (20–22) that are harmful to interpersonal relationships within the workplace to varying degrees (47–51). Precisely, as a dynamic process, interpersonal conflict consists of three essential elements: disagreement, interference, and negative emotions (52). It refers to incompatibility between the interacting parties (52, 53). The difference between workplace aggression and conflict is that formal refers to any physical or verbal specific actions that employees intentionally behave to hurt others (54). Obviously, although it has some similarities with interpersonal conflict, initiative and harmfulness are the main characteristics of aggression (55). Compared with interpersonal conflict, aggression is more harmful to the well-being of others and even organizations.

In this paper, we argue that ego depletion positively relates to interpersonal conflict and aggressive behaviors. First, individuals in a state of ego depletion are more likely to stuck in maladaptive cognition or lose effective regulation of their behaviors (56, 57). Therefore, owing to the decline of self-control capabilities, employees may vent their emotions by engaging in low-intensity workplace deviant behaviors (i.e., interpersonal conflict), even acting aggressively toward others. Second, individuals in a state of ego depletion are less likely to resist the impulse to violate work norms (58). In general, individuals take full consideration and weigh the conflicts between gaining self-interest and complying with work norms before acting (34). However, individuals in a state of ego depletion are unlikely to make trade-offs because of the lack of self-control resources. They prefer to follow their inner impulse even if these actions violate social norms (44). Therefore, those low in self-control resources are likely to act without thinking about others’ feelings or consequences, leading to interpersonal conflict and even physical and verbal aggression against others. Thus, we propose that:

*H2b*: Ego depletion is positively related to employee interpersonal conflict.

*H2c*: Ego depletion is positively related to employee aggression.

Combining the explanation of Hypotheses 1, 2a, 2b, and 2c, we propose that ego depletion is a potential mechanism mediating the relationship among COVID-related work changes, mental health, interpersonal conflict, and aggression. According to ego depletion theory, prior volitional behaviors that consume excessive resources may adversely affect the individual’s subsequent behavior (23). Therefore, employees need to devote excessive resources to assimilate to COVID-related work changes, which forces them to enter a state of ego depletion. At the same time, the state of ego depletion further hurts their mental health and increases inappropriate workplace behaviors (i.e., interpersonal conflict and aggression). Thus, we propose that:

*H3a*: Ego depletion mediates the relationship between COVID-related work changes and employees’ mental health.

*H3b*: Ego depletion mediates the relationship between COVID-related work changes and employees’ interpersonal conflict.

*H3c*: Ego depletion mediates the relationship between COVID-related work changes and employees’ aggression.

### Moderating effects of trait resilience

Trait resilience reflects an ability that assists individuals in adapting to stressful circumstances and recovering from loss, hardship, and adversity (24, 25). In the face of stress, trait resilience equips individuals with resources or energy to assess the hardship and stabilize their emotions (59) to protect better and construct their reservoir of resources. Previous research demonstrates that individuals with high resilience are more able to mitigate negative influences and cope with stress positively than those with low resilience (60, 61).

According to the ego depletion theory, individuals with different traits vary in their ability to mobilize and gather resources (62, 63). Hence, we argue that individuals with high trait resilience are less prone to fall into ego depletion when coping with COVID-related work changes versus those has low. First, individuals with high trait resilience can better mobilize resources to cope with change by shifting negative attention to the positive aspects of events (64, 65). A main reason for employees’ negative outcomes caused by the COVID-related work changes is that individuals cannot manage their own mental resources appropriately, so that they are tired to cope with the work changes and cannot maintain self-regulation. Thus, individuals with high trait resilience can cope with COVID-related changes effectively by consuming fewer self-control resources and are less likely to fall into a state of ego depletion. Conversely, individuals with lower trait resilience are more susceptible to shifting personal attention to the negative side of events and have difficulty coping with stress (61). Although they probably invest more resources to adapt to the change, it may have little effect or even more quickly lead to the depletion of self-control resources. Second, trait resilience enables individuals to seek out potential opportunities to access resources even when confronting severe adversity (64). Mitchell et al. (59) confirmed that individuals with varying trait resilience might make contrasting evaluations when faced with the same event. Individuals with high trait resilience tend to extract beneficial and valuable information from events and reject the negative aspects. This allows them to actively replenish their resource base even in the face of adversity (59, 60). Conversely, those low in trait resilience are likely overwhelmed by negative influences, resulting in excessive consumption of self-regulation resources. Hence, we hypothesize the following:

*H4*: Trait resilience moderates the relationship between COVID-related work changes and ego depletion, and the positive effect will be weaker when trait resilience is higher versus lower.

Previous research suggests that individuals with high trait resilience can actively search for a route in response to uncertain circumstances that do not lend themselves to planning, preparation, rationalization, or logical interpretation. (66, 67). Therefore, we propose that trait resilience can further moderate the effects of COVID-related work changes on employee mental health and workplace deviant behavior. Specifically, employees with higher trait resilience could better handle COVID-related work changes and still maintain the necessary resources. In doing so, abundant resources can provide high-trait resilience employees with the ability to better deal with negative emotions and control their behavior. On the contrary, individuals with low trait resilience are exhausted in their subsequent performance owing to the excessive resources consumed in response to COVID-related work changes, which may aggravate the negative impact of COVID-related work changes on employee psychology and behavior.

*H5a*: Trait resilience moderates the indirect relationship between COVID-related work changes and mental health, and the negative effect will be weaker when trait resilience is higher versus lower.

*H5b*: Trait resilience moderates the indirect relationships between COVID-related work changes and interpersonal conflict, and the negative effect will be weaker when trait resilience is higher versus lower.

*H5c*: Trait resilience moderates the indirect relationships between COVID-related work changes and aggression, and the negative effect will be weaker when trait resilience is higher versus lower.

## Methods

### Samples

During the COVID-19 pandemic, the front-line employees’ work environment, methods, and job contents were dramatically changed based on the modification and redesign of workflows. The manufacturing sector was one of the industries that severely affected by the pandemic and with constrict restrictions to cope with the spreading of the virus (68). To ensure the smooth operation of the economy amid the COVID-19, it is imperative for manufacturing enterprises to recall employees to restart production activities. Although companies have adopted a series of isolation measures to ensure the safety of front-line employees, these employees still suffer greater risk of infection than others. Furthermore, the majority of companies had laid off some workers for saving costs because they did not know when the market would recover and when migrant workers would be allowed to come back to work due to the travel restrictions. Such work changes and the concerned of being infected imposes substantial physical and psychological stress on employees. At the same time, the requirement to familiarize themselves with new technologies and environments in a short period can also significantly consume the energy of front-line employees. Therefore, we targeted our research on front-line workers engaged in manufacturing companies’ production, service, and logistics operations.

The sample of this study was front-line workers who worked in a large-scale Chinese manufacturing company with many subsidiaries. Most of the subsidiary companies are located in Shandong, Anhui, Sichuan, and Jiangsu. This research project was initiated in China in October 2022, in the immediate aftermath of the localized outbreak of COVID-19 in China. All of these subsidiaries were affected by the COVID-19 pandemic restriction. The cities where they are located reported infection cases during this study in October 2022. In compliance with China’s epidemic prevention policy, we conducted an online survey^1^ with the help of the HR department instead of issuing questionnaires through offline visits. In the recruitment process, we clarified the content, confidentiality, and voluntary nature of this study, to the participants. After completing the survey, we also offered them a prize in the form of an online lottery. Finally, we gathered 552 employees to participate in this study.

In line with Meade and Craig (69) and Huang et al. (70), we filtered out participants who chose the same options on most questions and completed the questionnaire in less than half the time to ensure the quality of the collected data. Finally, we received 536 valid questionnaires, accounting for 97.10% of the total sample size. Among these samples, 47.01% were male, and 52.99% were female; 31.34% were 36–45 years old, 26.12% were 46–55 years old, and 25.75% were 26–35 years old. In terms of educational level, 27.99% held an associate degree, 26.12% graduated from high school and 21.83% had a bachelor’s degree; 39.93% earned 5,000–7,500 Yuan per month, 21.27% earned 2,500–5,000 Yuan per month, and 14.37% earned 7,500–10,000 Yuan per month; 22.95% had worked in this organization for 2–3 years, 21.83% had worked for 1–2 years, and 19.22% had worked for 3–5 years.

### Measures

We adopted all the measurements in this study from previous research and translated them into Chinese following the back-translation procedure (71). Participants were required to rate the items with a 5-point Likert scale ranging from 1 = not at all to 5 = fully compliant. The specific measurement items of variables are shown in the Appendix.

### COVID-related work changes

We used an 8-item scale from Madero Gómez et al. (72) to assess the employees’ perceptions of the effect that COVID-19 has on their work (Cronbach’s α = 0.915). A representative item is “My workplace has had to modify its operational processes owing to the coronavirus.”

### Ego depletion

We used a 5-item scale from Twenge et al. (73) to measure ego depletion (Cronbach’s α = 0.877). A sample item is “My mind feels unfocused right now.”

### Mental health

We used a 5-item scale from Wu et al. (74) to measure mental health (Cronbach’s α = 0.916). A sample item is “I have been feeling emotionally stable lately.”

### Interpersonal conflict

We used a 4-item scale from Spector and Jex (75) to measure interpersonal conflict (Cronbach’s α = 0.821). A sample item is “Get into arguments with others at work.”

### Aggression

We used a 4-item scale from Stewart et al. (55) to measure aggression (Cronbach’s α = 0.888). A sample item is “I say something hurtful to someone at work.”

### Trait resilience

We used a 3-item scale from Smith et al. (76) to measure trait resilience (Cronbach’s α = 0.825). A sample item is “I usually come through difficult times with little trouble.”

### Control variables

We controlled the effects of gender, age, education level, monthly income (39), and years of employment to eliminate their possible confounding influence. Previous research has shown that job satisfaction serves as an effective predictor of psychological and behavioral changes (77, 78). To better demonstrate the effects of COVID-related work changes on employees, we adopted a 5-item scale from Judge, Locke, Durham, and Kluger (79) and controlled the effects of job satisfaction in all phases.

Meanwhile, we also controlled the relatively stable traits (i.e., emotional stability and resistance to change). Participants were required to rate their emotional stability using a 5-item scale (Cronbach’s α = 0.866) from Saucier (80) and their attitudes toward change by answering a 17-item scale (Cronbach’s α = 0.977) developed by Oreg et al. (81). As opposed to controlling the effects of emotional stability at all stages, resistance to change was only controlled in the path of influence on mediating variables.

## Results

We conducted a confirmatory factor analysis to confirm the discriminant validity of the hypothesized model using Mplus 8.1. As shown in Table 1, the fit indexes of the 9-factor model (χ^2^ = 1821.064, df = 1,448, χ^2^/df = 1.258, CFI = 0.982, TLI = 0.981, RMSEA = 0.022, SRMR = 0.029) offer a better fit for the collected data than any other models.

**Table 1:** Confirmatory factor analysis.

Model	χ^2^	df	*χ*^2^/df	CFI	TLI	RMSEA	SRMR
Nine-factor model: CWC, TR, ED, MH, IC, AG, RC, JS, ES	1,821.064	1,448	1.258	0.982	0.981	0.022	0.029
Eight-factor model: CWC + AG, ED, TR, MH, IC, RC, JS, ES	2,720.878	1,456	1.869	0.939	0.936	0.040	0.043
Seven-factor model: CWC + AG + IC, ED, TR, MH, RC, JS, ES	4,096.037	1,463	2.800	0.873	0.866	0.058	0.057
Six-factor model: CWC + AG + IC + MH, ED, TR, RC, JS, ES	4,591.417	1,469	3.126	0.850	0.842	0.063	0.061
Five-factor model: CWC + AG + IC + MH, JS + ES, ED, TR, RC	5,299.084	1,474	3.595	0.816	0.807	0.070	0.065
Four-factor model: CWC + AG + IC + MH + ED, JS + ES, TR, RC	6,226.674	1,478	4.212	0.771	0.762	0.077	0.071
Three-factor model: CWC + AG + IC + MH + ED + JS + ES, TR, RC	7,357.644	1,481	4.968	0.717	0.706	0.086	0.084
Two-factor model: CWC + AG + IC + MH + ED + JS + ES + TR, RC	7,835.125	1,483	5.283	0.694	0.682	0.089	0.086
One-factor model: CRWC + AG + IC + MH + ED + JS + ES + TR + RC	12,460.022	1,484	8.396	0.471	0.451	0.117	0.181

N = 536. CWC, COVID-related Work Changes; ED, Ego Depletion; TR, Trait Resilience; MH, Mental Health; IC, Interpersonal Conflict; AG, Aggression; RC, Resistance to Change; JS, Job Satisfaction; ES, Emotional Stability. Same for the following tables.

Table 2 summarizes the descriptive statistics and correlations of the study variables. COVID-related work changes are positively associated with ego depletion (*r* = 0.331, *p* < 0.01); ego depletion is negatively associated with mental health (*r* = −0.393, *p* < 0.01), and positively associated with interpersonal conflict (*r* = 0.355, *p* < 0.01) and aggression (*r* = 0.293, *p* < 0.01).

**Table 2:** Means, standard deviations, and correlations.

	Mean	SD	1	2	3	4	5	6	7	8	9	10	11	12	13	14
1. Gender	1.53	0.50	–													
2. Age	3.74	1.09	−0.010	–												
3. Education level	2.98	1.20	0.027	0.017	–											
4. Monthly income	2.99	1.16	0.044	−0.050	0.046	–										
5. Years of employment	3.27	1.52	0.007	−0.030	−0.023	−0.040										
6. RC	2.71	0.96	0.018	0.103*	−0.006	−0.002	0.019	**(0.977)**								
7. JSA	3.33	0.69	0.080	−0.094*	−0.019	−0.037	0.007	−0.105*	**(0.854)**							
8. ES	3.40	0.68	0.108*	−0.065	0.010	0.007	0.012	−0.168**	0.409**	**(0.866)**						
9. CWC	2.40	0.81	−0.050	0.011	0.023	0.039	−0.031	0.167**	−0.219**	−0.244**	**(0.915)**					
10. ED	2.48	0.80	−0.016	0.001	−0.015	0.036	−0.048	0.123**	−0.200**	−0.291**	0.331**	**(0.877)**				
11. TR	3.33	0.75	0.094*	0.008	−0.008	−0.029	0.003	−0.130**	0.295**	0.279**	−0.246**	−0.234**	**(0.825)**			
12. MH	3.64	0.83	0.016	0.106*	−0.012	−0.013	0.080	−0.132**	0.283**	0.342**	−0.387**	−0.393**	0.279**	**(0.916)**		
13. IC	3.03	0.69	−0.022	0.003	0.029	0.045	0.015	0.126**	−0.243**	−0.308**	0.422**	0.355**	−0.251**	−0.331**	**(0.821)**	
14. AG	2.21	0.79	−0.016	−0.102*	0.059	0.025	−0.013	0.108*	−0.211**	−0.260**	0.448**	0.293**	−0.220**	−0.423**	0.333**	**(0.888)**

*N* = 536. Same for the following tables. Internal consistent reliability (alpha) coefficients are shown along the diagonal in bold italics. Gender, 1 = male, 2 = female. Age, 1 = under 18 years old, 2 = 18–25 years old, 3 = 26–35 years old, 4 = 36–45 years old, 5 = 46–55 years old, 6 = over 56 years old. Education level, 1 = junior high school degree or below, 2 = high school, 3 = associate degree, 4 = bachelor degree, 5 = master degree or above. Monthly income, 1 = under 2,500 Yuan, 2 = 2,500–5,000 Yuan, 3 = 5,000–7,500 Yuan, 4 = 7,500–10,000 Yuan, 5 = over 10,000 Yuan. Years of employment, 1 = below 1 year, 2 = 1–2 years, 3 = 2–3 years, 4 = 3–5 years, 5 = 5–10 years, 6 = over 10 years. **p < 0.01, *p < 0.05.

Table 3 displays the results for the direct, indirect, and moderate hypotheses and demonstrates their bootstrapped estimates, standard errors, and confidence intervals. COVID-related work changes significantly and positively affect employees’ ego depletion (*β* = 0.229, *p* < 0.001), which supports Hypothesis 1. Meanwhile, consistent with Hypotheses 2a, 2b, and 2c, ego depletion has a significant and negative impact on mental health (*β* = −0.313, *p* < 0.001) and has a significant and negative effect on interpersonal conflict (*β* = 0.241, *p* < 0.001) and aggression (*β* = 0.224, *p* < 0.001). Consistent with Hypotheses 3a, 3b, and 3c, ego depletion mediates the relationships among COVID-related work changes and mental health, COVID-related work changes and interpersonal conflict, and COVID-related work changes and aggression. Specifically, for mental health, the indirect effect is −0.072 (95% CI = [−0.109, −0.043]); For interpersonal conflict, the indirect effect is 0.055 (95% CI = [0.032, 0.087]); For aggression, the indirect effect is 0.051 (95% CI = [0.026, 0.084]).

**Table 3:** Summary of direct, indirect, and interaction effects.

Paths	Estimates	S.E.	95% CI	Significance
Direct effects				
COVID-related Work Changes → Ego Depletion	0.229	0.039	[0.154, 0.308]	*p* < 0.001
Ego Depletion →Mental Health	−0.313	0.042	[−0.396, −0.228]	*p* < 0.001
Ego Depletion →Interpersonal Conflict	0.241	0.035	[0.175, 0.311]	*p* < 0.001
Ego Depletion →Aggression	0.224	0.042	[0.139, 0.306]	*p* < 0.001
Indirect effects				
COVID-related Work Changes → Ego Depletion→ Mental Health	−0.072	0.017	[−0.109, −0.043]	*p* < 0.001
COVID-related Work Changes → Ego Depletion→ Interpersonal Conflict	0.055	0.014	[0.032, 0.087]	*p* < 0.001
COVID-related Work Changes → Ego Depletion→ Aggression	0.051	0.015	[0.026, 0.084]	*p* < 0.001
Moderate effects				
COVID-related Work Changes * Trait Resilience →Ego Depletion	−0.115	0.053	[−0.221, −0.010]	*p* < 0.050

*N* = 536. Estimates, bootstrapped estimate; SE, standard error; LL, lower level; UL, upper level; CI, confidence interval. Values for quantitative moderators are the plus/minus one SD from the mean. Same for the following tables.

**Figure 2:**
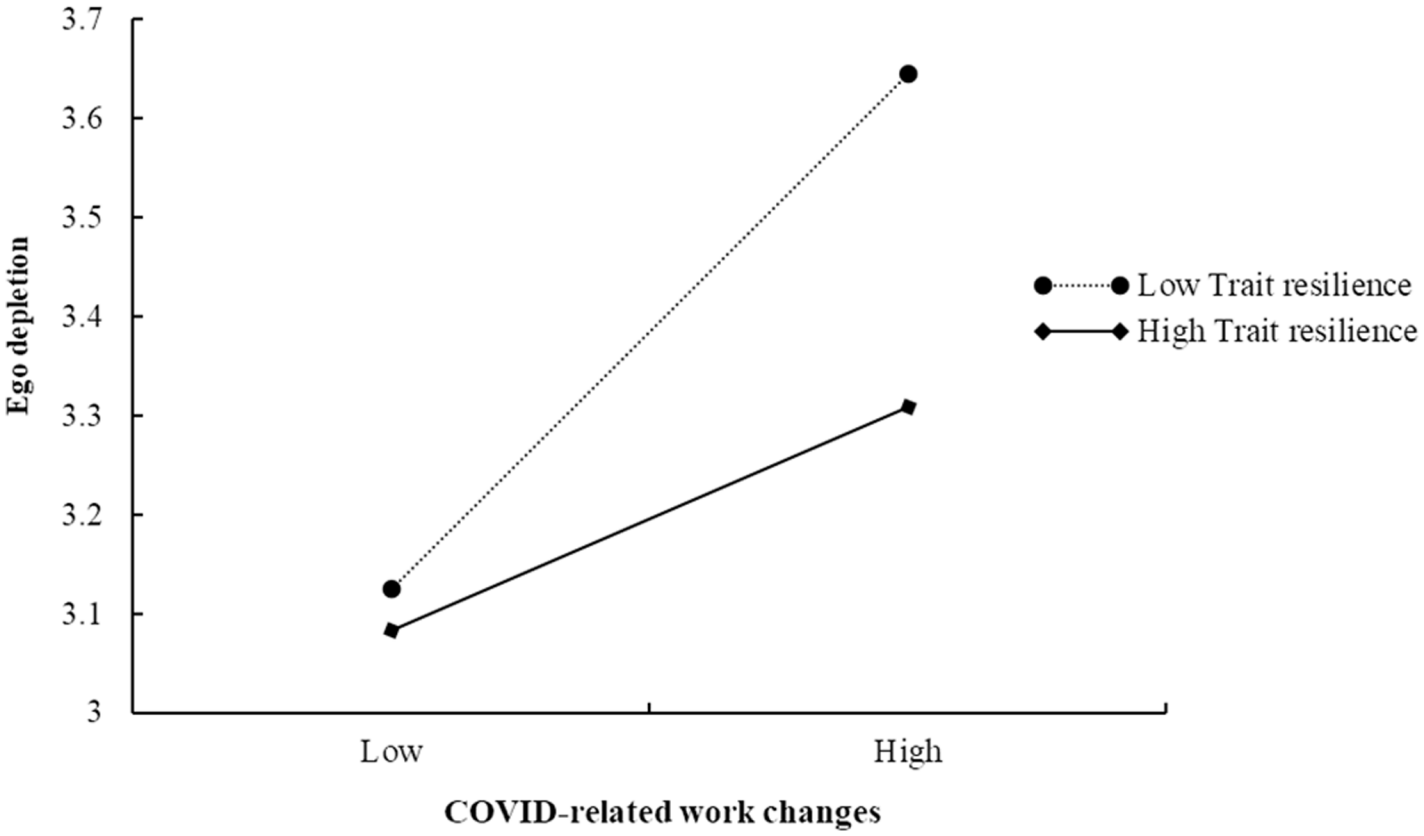
The moderating effect of trait resilience on the relationship between COVID-related work changes and ego depletion.

Table 3 also reveals that the interaction between COVID-related work changes and trait resilience is negatively related to ego depletion (*β* = −0.115, 95% CI = [−0.221, −0.010]). The finding demonstrates that the positive effect of COVID-related work changes on ego depletion significantly varies for individuals with different levels of resistance to change, as shown in Figure 2. Simple slope analysis indicates that the positive effect of COVID-related work changes on ego depletion is weaker for individuals with high trait resilience versus low trait resilience. Thus, Hypothesis 4 is supported.

Table 4 displays conditional indirect effects at values of trait resilience. The results indicate that trait resilience moderates the indirect effects of COVID-related work changes on mental health, interpersonal conflict, and aggression through ego depletion. Ego depletion has a weaker mediation effect on the relationship between COVID-related work changes and mental health for employees with high-level trait resilience (i.e., conditional mediation effect = −0.036, 95% CI = [−0.086, 0.008]) than for employees with low-level trait resilience (i.e., conditional mediation effect = −0.108, 95% CI = [−0.161, − 0.062]), consistent with Hypothesis 5a. Additionally, the difference in these two effects is 0.036 (95% CI = [0.004. 0.074]). Supporting Hypothesis 5b, ego depletion has a weaker mediation effect on the relationship between COVID-related work changes and interpersonal conflict for employees with high-level trait resilience (i.e., conditional mediation effect = 0.027, 95% CI = [−0.006, 0.069]) compared to employees with low-level trait resilience (i.e., conditional mediation effect = 0.083, 95% CI = [0.050, 0.125]), and the difference between these two effects is −0.028 (95% CI = [−0.056, −0.005]). Supporting Hypothesis 5c, ego depletion has a weaker mediation effect on the relationship between COVID-related work changes and aggression for employees with high-level trait resilience (i.e., conditional mediation effect = 0.026, 95% CI = [−0.005, 0.067]) compared to employees with low-level trait resilience (i.e., conditional mediation effect = 0.077, 95% CI = [0.043, 0.121]), and the difference between these two effects is −0.026 (95% CI = [−0.053, −0.005]).

**Table 4:** Summary of conditional indirect effects at values of trait resilience.

Level	Estimates	S.E.	95% CI
Conditional indirect effects at values of Trait Resilience (COVID-related Work Changes → Ego Depletion→ Mental Health)			
−1 SD	−0.108	0.025	[−0.161, −0.062]
+1 SD	−0.036	0.024	[−0.086, 0.008]
Difference	0.036	0.018	[0.004, 0.074]
Conditional indirect effects at values of Trait Resilience (COVID-related Work Changes → Ego Depletion→ Interpersonal Conflict)			
−1 SD	0.083	0.019	[0.050, 0.125]
+1 SD	0.027	0.019	[−0.006, 0.069]
Difference	−0.028	0.013	[−0.056, −0.005]
Conditional indirect effects at values of Trait Resilience (COVID-related Work Changes → Ego Depletion→ Aggression)			
−1 SD	0.077	0.020	[0.043, 0.121]
+1 SD	0.026	0.018	[−0.005, 0.067]
Difference	−0.026	0.012	[−0.053, −0.005]

## Discussion

Based on ego depletion theory, we constructed a moderated mediation model to explain how and when COVID-related work changes can influence employees’ mental health and their workplace deviant behavior. Specifically, we explained the mediation role of employees’ ego depletion and the moderate effect of trait resilience. Based on the 536 samples collected from a large manufacturing company, we found that COVID-related work change harms employees’ mental health *via* ego depletion. This finding confirms Trógolo et al.’s (82) conclusion that COVID-related work change increases psychological stress, which might harm employees’ health. Furthermore, our paper also indicated that COVID-related work changes exert a positive and significant effect on interpersonal conflict and aggression. The result validates Leslie et al.’s (83) survey that COVID-related work change might increase workplace deviant behavior among employees. Additionally, we discovered that trait resilience could weaken the promoting effect of COVID-related work changes on ego depletion and negatively adjust the mediating effect of COVID-related work changes on employees’ mental health and deviant workplace behavior through ego depletion.

### Theoretical implications

There are several implications of this study. The first contribution lies in extending the literature on work change in the context of COVID-19 by exploring the effects of COVID-related work changes on employees’ mental health and workplace deviant behavior. The majority of previous researchers studied the relationship between a specific aspect of COVID-related work changes and employees’ attitudes and outcomes, such as working from home (72, 84), virtual teams (31), and virtual meetings (32). However, COVID-related work changes encompass workplace changes, work characteristics, and the workforce (85). As such, it is necessary to conduct a more comprehensive study to explore work change amid COVID-19 and its effect on employees. In our research, we discovered work changes in a broad-scope overview and enriched the research perspective of COVID-related work changes.

Second, this study revealed the underlying mechanism that could explain the influence of COVID-related work changes on employees’ mental health and workplace deviant behavior by highlighting the mediating effect of ego depletion. Previous researchers have found that workplace change may lead to work–family conflict, thus affecting the mental health and work performance of employees during COVID-19 (9, 82). However, relatively few researchers have explored COVID-related work changes’ impact on employees’ psychology and behavior and how this impact occurs. In this study, we constructed a model of how COVID-related work changes affect employees’ mental health and deviant workplace behavior through ego depletion, which can better clarify its mechanism.

Third, this study further answers the question of under which conditions COVID-related work changes may have stronger or weaker effects on employees’ cognition and behavior. The importance of individual trait resilience in positively responding to the COVID-19 crisis is attracting more research attention (86, 87), and we have reason to believe that trait resilience plays a positive moderating role in the mechanism of the negative impact of work change on employees during COVID-19. Surprisingly, no specific studies concern the moderating role of trait resilience. Thus, we examined the moderating effect of trait resilience on the relationship between COVID-related work changes and employees’ mental health and deviant workplace behavior to study employees’ mental health and deviant workplace behavior. In doing so, this study provides a complete picture for understanding the effect of COVID-related work changes on employees’ mental health and deviant workplace behavior.

### Practical implications

This study also provides some practical insights for managers. First, our research findings confirmed that COVID-related work changes would impair employees’ mental health and cause them to engage in interpersonal conflict and aggression. Therefore, our study provide hint for managers to understand the causes of employees’ mental health problems and inappropriate workplace behavior in the organization. Only by understanding the root of the problem can take the correct actions to solve the issue. For example, managers could build positive organizational climate to keep employees’ morale up so that employees will no longer worry about the related changes in their work. Also, organizations need to provide the necessary staff training to strengthen their work technical capacity and thus enhance their confidence in coping with work changes. In addition, managers should take appropriate actions in intervening in interpersonal conflicts and even aggressive behaviors between employees. At this point, managers should pay more attention to the mental status of employees and communicate with both parties to facilitate the resolution of their conflict.

Second, in this paper, we deemed that employees’ emotional and behavioral dysregulation is caused by excessive consumption of self-control resources when dealing with work changes. During this special period, employees may overexert themselves owing to the lack of self-control resources. In this case, it is no longer appropriate for managers to insist on dictatorial leadership, but should consider cultivating a democratic management style that facilitates employees’ regaining a sense of control over their work to overcome the negative psychological impact. Meanwhile, as the outbreak situation improves, managers should develop more flexible management forms (i.e., advice seeking, providing more feedback) to give employees some autonomy in their work, which improves the efficiency of organizational operations to a certain extent and helps employees recover from a state of self-attrition.

Third, this study indicated that high trait resilience could effectively weaken the negative effect of COVID-related work changes on employees’ mental health and has a positive effect on workplace deviant behavior. Hence, during the COVID-19 pandemic, we suggest that organizations should pay attention to the trait resilience of employees. On the one hand, managers can stimulate employees’ trait resilience by establishing reward and punishment system for boosting adaptive performance which is used to measure the responsiveness to changing job requirements (88). On the other hand, leaders should encourage employees to internalize organizational values to improve their trait resilience. Turning work initiative into an internal driving force can help change employees’ perception of work changes from stress to challenge. In doing so, their coping attitude toward COVID-19-related work changes would change from negative to positive.

### Limitations and future research

Although this study has the aforementioned theoretical and practical implications, there are still some limitations. First, although the current research demonstrates the impact of COVID-related work changes on employees’ mental health and workplace deviant behavior, our research design is cross-sectional, limiting our causality inference. Future researchers should explore whether work change always hurts employees’ recognition and behaviors by utilizing a longitudinal design or multi-wave data.

Second, this study revealed the underlying mechanism through which COVID-related work changes could damage employee mental health and workplace behavior from the perspective of self-control resources. Future researchers could further examine the effects of COVID-related work changes on employees from other perspectives and reveal the other potential paths. For instance, based on the appraisal theory of stress, scholars could examine how the differences in individuals’ subjective assessments of COVID-related work changes affect employees’ behavioral and psychological outcomes.

Finally, this research was conducted only in China, which limits the generalizability of the results to some degree. Future researchers could examine whether work change influences employees’ mental health and deviant workplace behavior through ego depletion in other countries, particularly in developed countries with entirely different social cultures from China.

## Data availability statement

The raw data supporting the conclusions of this article will be made available by the authors, without undue reservation.

## Author contributions

CH, RZ, and XiyL contributed to conception and design of the study. CH organized the database. RZ and XiyL performed the statistical analysis. HL and GH wrote the first draft of the manuscript. SL and XiaL wrote sections of the manuscript. CH, XiyL, and XW contributed to manuscript revision, read, and approved the submitted version.

## Conflict of interest

The authors declare that the research was conducted in the absence of any commercial or financial relationships that could be construed as a potential conflict of interest.

## Publisher’s note

All claims expressed in this article are solely those of the authors and do not necessarily represent those of their affiliated organizations, or those of the publisher, the editors and the reviewers. Any product that may be evaluated in this article, or claim that may be made by its manufacturer, is not guaranteed or endorsed by the publisher.

## Appendix

Factor structure

COVID-related work changes [Madero Gómez et al. (72)]

1. The organization where I work has been affected negatively by the emergence of the coronavirus.
2. Production or service processes of the organization where I work will be affected in the next couple of months by the coronavirus.
3. The coronavirus has put my workplace’s operations at risk.
4. The coronavirus will be a reason for more absenteeism than normal in my workplace.
5. Imports of raw material in my organization have been negatively affected by the coronavirus.
6. My organization’s operations have been negatively affected by the coronavirus.
7. My workplace has had to modify its operational processes due to the coronavirus.
8. My workplace has had to modify its travel policies and guidelines due to the coronavirus.

Ego depletion [Twenge et al. (73)]

1. I feel drained.
2. My mind feels unfocused right now.
3. Right now, it would take a lot of effort for me to concentrate on something.
4. My mental energy is running low.
5. I feel like my willpower is gone.

Mental health [Wu et al. (74)]

1. I have been feeling positive lately.
2. I have been feeling emotionally stable lately.
3. I have been feeling satisfied with life lately.
4. I have been feeling life had been interesting lately.
5. I have been feeling everything to look forward to lately.

Interpersonal conflict [Spector and Jex (75)]

1. Get into arguments with others at work.
2. Other people yell at you at work.
3. People rude to you at work.
4. People do nasty things to you at work.

Aggression [Stewart et al. (55)]

1. Said something hurtful to someone at work.
2. Acted rudely toward someone at work.
3. Lost their temper while at work.
4. Made fun of someone at work.

Trait resilience [Smith et al. (76)]

1. I tend to bounce back quickly after hard times.
2. It does not take me long to recover from a stressful event.
3. I usually come through difficult times with little trouble.

## Footnotes

^1^ https://www.wjx.cn/
