# Corrigendum: The virus made me lose control: the impact of COVID-related work changes on employees' mental health, aggression, and interpersonal conflict

**DOI:** 10.3389/fpubh.2023.1223030

**Published:** 2023-07-06

**Authors:** Changlin Han, Ruyi Zhang, Xiyao Liu, Xueling Wang, Xiaotong Liu

**Affiliations:** ^1^School of Business, Qingdao University, Qingdao, Shandong, China; ^2^Student Affairs Department, Shandong University, Jinan, Shandong, China

**Keywords:** COVID-related work changes, ego depletion, mental health, interpersonal conflict, aggression, trait resilience

In the published article, there was an error. In the **Author Contributions**, author Xiaotong Liu was erroneously excluded.

A correction has been made to **Author Contributions**, paragraph 1. This sentence previously stated:

“CH, RZ, and XiyL contributed to conception and design of the study. CH organized the database. RZ and XiyL performed the statistical analysis. HL and GH wrote the first draft of the manuscript. SL and XiaL wrote sections of the manuscript. CH, XiyL, and XW contributed to manuscript revision, read, and approved the submitted version.”

The corrected sentence appears below:

“CH, RZ, and XiyL contributed to the conception and design of the study. CH organized the database. RZ and XiyL performed the statistical analysis. XiaL wrote sections of the manuscript. CH, XiaL, XiyL, and XW contributed to manuscript revision, read, and approved the submitted version. All authors contributed to the article and approved the submitted version.”

The authors apologize for this error and state that this does not change the scientific conclusions of the article in any way. The original article has been updated.

